# Scene grammar shapes the way we interact with objects, strengthens memories, and speeds search

**DOI:** 10.1038/s41598-017-16739-x

**Published:** 2017-11-28

**Authors:** Dejan Draschkow, Melissa L.-H. Võ

**Affiliations:** Scene Grammar Lab, Johann Wolfgang Goethe-Universität, Frankfurt, Germany

## Abstract

Predictions of environmental rules (here referred to as “scene grammar”) can come in different forms: seeing a toilet in a living room would violate *semantic* predictions, while finding a toilet brush next to the toothpaste would violate *syntactic* predictions. The existence of such predictions has usually been investigated by showing observers images containing such grammatical violations. Conversely, the generative process of creating an environment according to one’s scene grammar and its effects on behavior and memory has received little attention. In a virtual reality paradigm, we either instructed participants to arrange objects according to their scene grammar or against it. Subsequently, participants’ memory for the arrangements was probed using a surprise recall (Exp1), or repeated search (Exp2) task. As a result, participants’ construction behavior showed strategic use of larger, static objects to *anchor* the location of smaller objects which are generally the goals of everyday actions. Further analysis of this scene construction data revealed possible commonalities between the rules governing word usage in language and object usage in naturalistic environments. Taken together, we revealed some of the building blocks of scene grammar necessary for efficient behavior, which differentially influence how we interact with objects and what we remember about scenes.

## Introduction

Although our world is complex, it adheres to certain rules. These are rules which we learn and continuously update throughout our lives. For instance, we realized early on that some objects belong to certain contexts (e.g. pot goes in the kitchen). In addition to these *semantic* rules, *syntactic* rules tell us that objects need a surface to rest on and that some objects have very defined spatial relations, e.g. the pot rests on a stove. Therefore, scenes, similar to language, constitute rule-governed arrangements of their elements — objects and words, respectively^1,2^. Knowledge of scene-based rules – which we have come to call *scene grammar* ^3,4^ – allows us to easily anticipate the identity of objects within a scene, alleviating the computational load of perceptual processes^5,6^. Therefore, a stronger understanding of scene grammar is key to understanding the efficiency of the perceptual process. Higher-level visual predictions have usually been investigated by showing observers images of environments in which these predictions are violated, e.g. a soap hovering above a sink (syntax) or football in the fridge (semantics). This work has shown that violations of scene grammar can lead to slower and less accurate identification of objects^2,5,7^ and are accompanied by distinct neural components^4,8–10^. There is evidence that scene grammar can facilitate tasks such as visual search by guiding eye movements^3,11–13^ and by strengthening the formation of visual representations^14,15^. Violations of one’s scene grammar usually result in longer and more frequent fixations on the critical objects^16–18^, and can impede visual search^12,19^. Further, by increasing the availability of contextual information, participants rely less on recently formed memory representations and more on a generalized scene grammar to guide search^20^.

The importance of scene grammar for guiding behavior in real-world environments seems apparent. However, little is known about the generative process of creating meaningful contexts. How does, for example, creating environments that adhere either more or less to common scene grammar affect behavior and memory? Does a chaotic environment – even if the chaos is self-generated – affect cognition therein? Methodological constraints have thus far made it very difficult for the field to study the influence of scene grammar during the construction of our environments as well as subsequent physical interactions therein – in natural behavior, however, we not only interact with our environment with our gaze, but also physically handle objects. We utilized a virtual reality paradigm to overcome these constraints and answer some of these fundamental questions. In order to understand the way we represent natural environments it is important to consider ecologically valid settings in which participants are free to move around and perform a natural task^21–28^, not least because spatial memory changes its reference frame when moving from static to dynamic tasks^23,29,30^. For the current study, we either instructed participants to arrange objects in a meaningful way within a virtual reality environment (e.g. placing the pot on the stove), or to specifically violate scene grammar. Subsequently, participants’ memory for the arrangements was probed using a surprise explicit recall task (Experiment 1), or a repeated search task (Experiment 2).

In order to understand the regularities of our visual environment, we need to identify its building blocks. Not all objects are created equal within a scene, so identifying elements of a scene which contribute differently to the predictions we generate about the scene as a whole as well as other objects is crucial for understanding scene grammar. Some objects can support the identification of a scene better than others – a pan usually suggest that we are in a kitchen, but a wallet could be anywhere^31^. Further, expected spatial relations between objects can facilitate search^19,32^ and object recognition^5^. To investigate if the influence of scene grammar on current and future behavior is mediated by different components of a scene, we distinguished local and global objects. We consider *local* objects to be objects which are generally movable, the goals of our action in everyday life, and considered to be *acted upon* ^27,33,34^. *Global* objects, on the other hand, are usually static elements, which we hypothesize to function as “anchors” for spatial predictions regarding other objects within a scene. For instance, in indoor scenes, anchors like desks, beds, or stoves might strongly influence spatial predictions regarding the computer, the pillow, or the pot. We aim to test this hypothesis by providing empirical evidence for differences between these components of a scene.

Finally, many of our current and future interactions with scenes depend on the knowledge we have gained from past interactions with similar environments. Previous research has concentrated on the consequences of that knowledge. The current study, instead, now specifically addresses the generative process of spatial arrangements and thus allows us to draw parallels to another form of generative behavior – the use of words. Uncovering similarities to basic rules of language generation can provide the first step towards a comprehensive understanding of commonalities between scenes perception and language processing. One of the most basic and puzzling facts of texts is that word occurrences can be described by a mathematically simple power law postulating that *few* high frequency words (.g., “the” and “of” in this paper) and *many* low frequency words (e.g. “badger”, “snout”, “quinoa”, etc.) will make up any given text^35–37^. That is, 80% of any given text is made up of the same 20% of highly frequent words. To investigate possible commonalities between systematic occurrences of words in language and systematic occurrences of objects in naturalistic environments, we calculated frequency distributions of object pairs positioned in close proximity across participants.

In the following sections, we will provide insights regarding the building blocks of scene grammar as it unfolds during scene construction, how scene grammar then influences the way we interact with objects and what we remember about a scene, and finally provide further hints for systematic commonalities between occurrences of words in language and the spatial arrangement of naturalistic environments.

## Results

### Experiment 1

In the first study we investigated how arranging environments according to – or in direct violation of – our predictions influence our behavior and extant memory representations and how these effects are modulated by the different building blocks of scene grammar.

The experimental procedure consisted of two phases – the build phase and the recall phase. During both phases‚ participants were instructed to arrange a fixed number of 15 objects in each room either according (consistent condition) or against (inconsistent condition) their expectations regarding the usual setup of the room category. After completing the arrangement of the 16 rooms in the build phase, participants were informed of a subsequent surprise recall test. In the *recall phase*, participants had to reconstruct all the rooms exactly as they had during the build phase, i.e. they were instructed to place the individual objects in the same locations in which they had placed them before. The experimental factors in Experiment 1 were Consistency (consistent vs. inconsistent), Object type (global vs. local), and Experimental Phase (build vs. recall). The results of the individual analysis procedures are summarized in Tables 1 and 2.

**Table 1:** Results of the linear mixed-model for grab duration including estimated regression coefficients together with the *t* statistic, as well as a Tukey corrected break down of significant interactions (left columns).

	Grab duration LMM			Grab order ANOVA			
	Estimate	*T*		df	*F*	*p*	*η* ^2^_*G*_
(Intercept)	0.020	0.293					
Condition (con vs. incon)	−0.046	−3.323		1,9	0.543	0.480	0.001
Object type (global vs. local)	−0.148	−8.442		1,9	280.265	0.001	0.932
Phase (building vs. recall)	0.103	−5.929		1,9	0.865	0.377	0.002
Condition × Object type	0.035	5.983		1,9	1.445	0.260	0.019
Condition × Phase	0.004	0.733		1,9	1.622	0.235	0.004
Object type × Phase	0.010	1.770		1,9	0.135	0.722	0.002
Condition × Object type × Phase	0.002	0.291		1,9	3.684	0.087	0.064
	**Tukey contrasts of LMM interaction**						
	**Estimate**	***Z***	***p***				
con (global) vs. incon (global)	−0.022	−0.737	0.882				
con (global) vs. con (local)	−0.225	−6.116	0.001				
con (global) vs. incon (local)	−0.389	−7.287	0.001				
incon (global) vs. con (local)	−0.203	−5.948	0.001				
incon (global) vs. incon (local)	−0.366	−9.877	0.001				
con (local) vs. incon (local)	−0.163	−5.344	0.001				

On the right, the statistics of the ANOVA for mean grab order are listed.

**Table 2:** Results of the simple linear mixed-model for location accuracy including estimated regression coefficients together with the *t* statistic (left columns).

	Recall LMM		Recall LMM * covariates	
	Estimate	*t*	Estimate	*t*
(Intercept)	−1.007	−12.764	−0.710	−6.060
Condition (con vs. incon)	−0.390	−8.028	−0.364	−4.094
Object type (global vs. local)	−0.192	−6.251	0.057	0.714
Gaze duration (log)			−0.118	−3.293
Grab duration (log)			−0.012	−0.281
Condition × Object type	−0.022	−1.036	0.019	0.251
Condition × Gaze duration (log)			−0.003	−0.105
Object type × Gaze duration (log)			−0.087	−2.748
Condition × Object type × Gaze duration (log)			−0.019	−0.626

On the right, the results of the LMM with gaze and grab duration as covariates.

#### The impact of consistency and object type on object interaction duration and order

We know from previous research that violations of one’s scene grammar usually result in longer and more frequent fixations on the critical objects^16–18^, which has been interpreted as increased processing of the inconsistent object. But in natural behavior observing or finding an object is usually just means to an end – the end being interactions with that object. Our paradigm allowed us to use object interaction behavior as a measure of processing time. Table 1 summarizes the results of the linear mixed-effects model for grab duration (details can found in the Data Analysis section, as well as in the analysis scripts).

**Figure 1:**
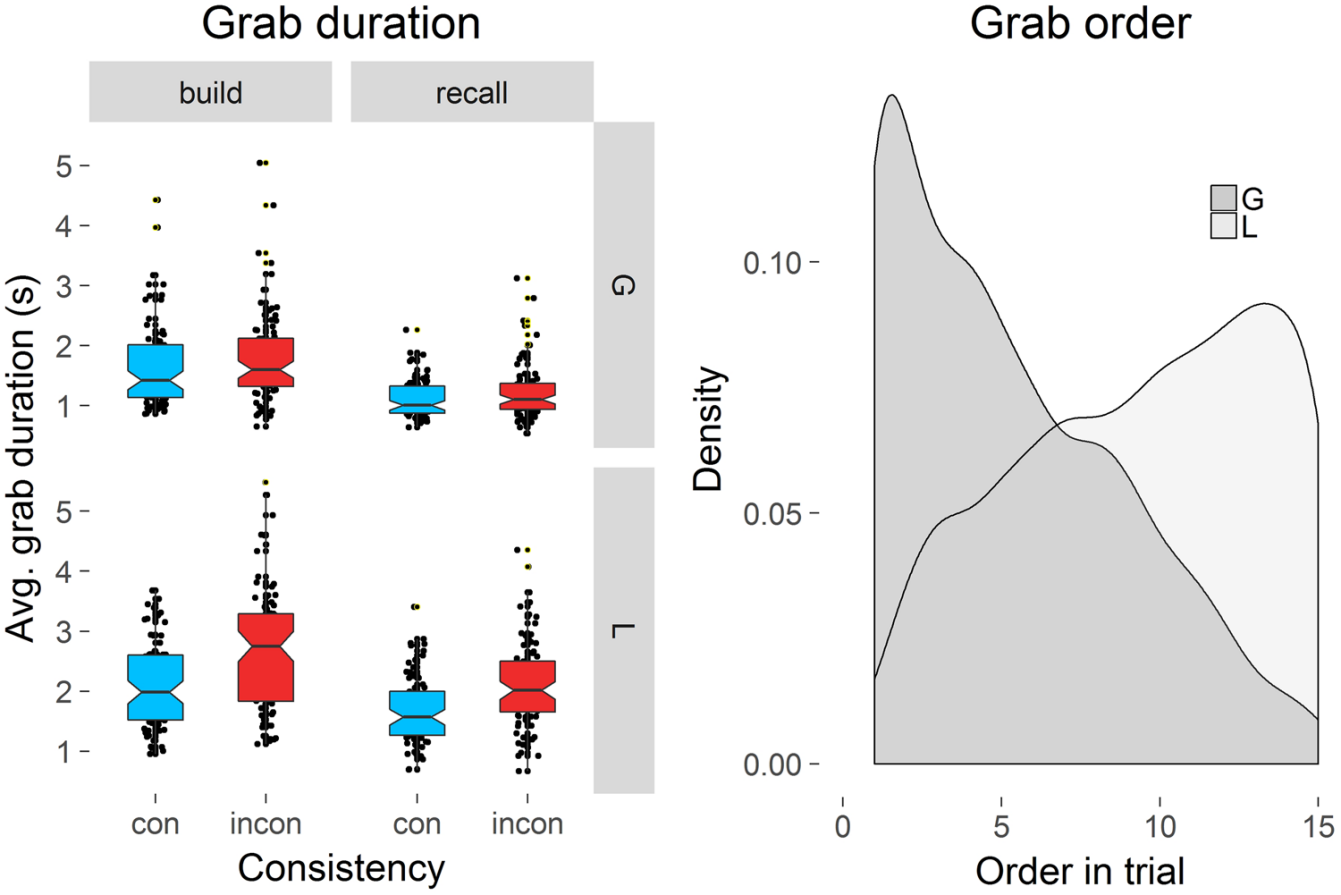
The effects of Consistency (consistent = con vs. inconsistent = incon), Object type (global = G vs. local = L), and experimental Phase (build vs. recall) on the grab duration (left). The central mark is the median of each boxplot. The notches indicate 95% confidence intervals for the medians. The right graph depicts computed density estimates (Gaussian smoothing kernel) (y-axis) for first object grabs during a trial (x-axis) as a function of Object type (global = G vs. local = L).

There was a main effect of Consistency, as objects were grabbed longer on average in the inconsistent compared to the consistent condition (Fig. 1). The main effects of Object type and Phase on grab duration were also significant, with local objects being handled longer than global, and grab durations in the build phase being longer than in the recall phase. The significant interaction between Consistency and Object type showed that the grab duration of global objects (e.g. a fridge) was not significantly affected by Consistency, whereas local objects (e.g. a toaster) were grabbed longer in the inconsistent compared to consistent condition. A repeated measures ANOVA on the mean grab order of objects revealed that global objects were handled earlier in the trial compared to their local counterparts. The right graph of Fig. 1 shows the probability of grabbing a global object for the first time decreased with every next object, whereas the probability increased for local objects.

#### Memory performance

To differentiate between subjects’ specific memories and successful guessing we estimated a baseline for memory performance by using a between-subject measure of location accuracy. That is, for each room arranged by each participant in the build phase of the experiment we randomly chose another participant and calculated the distances between the three-dimensional coordinates of the centers of identical objects within the same room. This measure not only provides us with a reliable performance baseline, but also gives us a first notion about some commonalities in scene grammar *across* participants.

**Figure 2:**
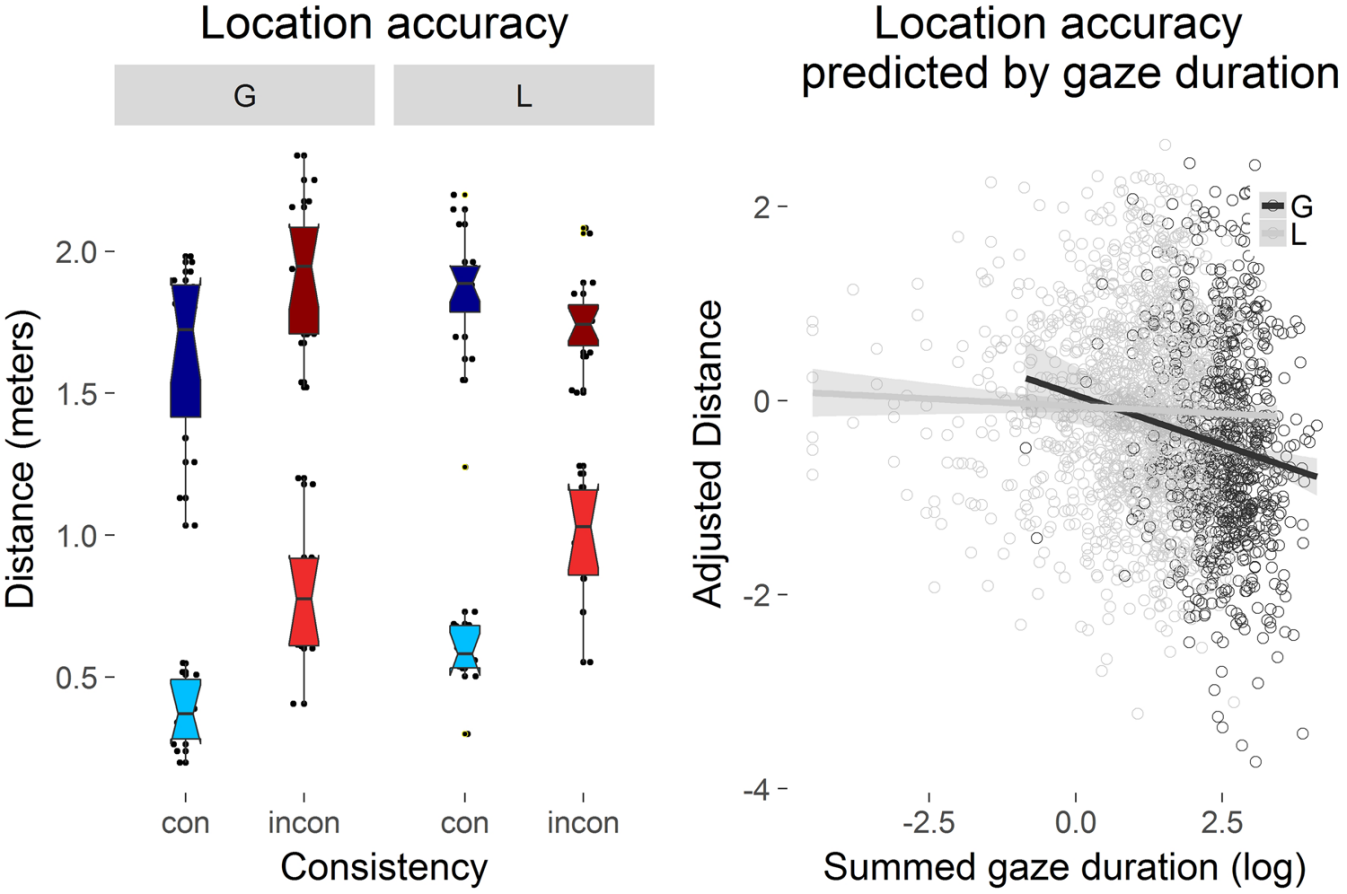
The effects of Consistency (consistent = con vs. inconsistent = incon) and Object type (global = G vs. local = L) on location accuracy measured in meters distance (left). Lower values indicate better location memory performance. The bright colored box plots represent the actual empirical data, whereas the dark colored box plots represent the cross-participant baseline estimation. The central mark is the median of each boxplot. The notches indicate 95% confidence intervals for the medians. The right graph displays partial effects (thus “adjusted”). Distance on the y-axis against log transformed gaze duration per object as a function of Object type (global = G vs. local = L). Shaded areas represent 95% confidence intervals.

Initially we conducted an F2-ANOVA on the performance baseline with Consistency and Object Type as factors. Neither factor showed a significant main effect, Consistency, *F*(1, 15) = 1.8, *p* = 0.19, *η* ^2^_*G*_ = 0.04 and Object Type, *F*(1, 15) = 0.41, *p* = 0.53, *η* ^2^_*G*_ = 0.004 (Fig. 2, left). However, there was a significant interaction, *F*(1, 15) = 14.2, *p* = 0.002, *η* ^2^_*G*_ = 0.1. A paired sample *t*-test revealed that the mean distance of the baseline between consistent global objects was less than between inconsistent global objects, *t*(15) = −2.68, *p* = 0.017, Cohen’s *d* = 0.67, confirming the intuition that predictions regarding global scene structure were more similar between participants in the consistent condition, compared to the inconsistent condition. Note that this was not true for local elements in the environment, *t*(15) = 1.46, *p* = 0.165, Cohen’s *d* = 0.365.

While participants’ overall recall performance was significantly better than baseline, *t*(75.02) = 19.26, *p* < 0.001, Cohen’s *d* = 3.94, consistent objects were recalled more accurately than inconsistent objects (Fig. 2, left). The distance between the original and the recalled location of global objects was less than that of local objects.

#### Predicting location memory

In a second memory performance model, we included summed grab durations as well as summed gaze durations for each object from the build phase as separate covariates (Fig. 2, right). Interactions with covariates which did not significantly improve the fit of the data were removed from the model and are not included in Table 2. Data points with missing values were removed, leading to a removal of 2.4% of the data. There was a main effect of Gaze Duration and a significant interaction between Gaze Duration and Object type, indicating that in comparison to memory for local objects, memory for global objects profited from longer viewing time (Table 2, right).

### Experiment 2

In Experiment 2, we aimed at replicating the effects of scene grammar violations on ongoing behavior and object interactions, as the experimental procedure of the build phase was identical to Experiment 1. Further, we wanted to expand the findings of the modulatory role of global and local objects to a different memory performance assessment – subsequent search, rather than the recall procedure of Experiment 1. Violations of one’s scene grammar can impede visual search^12,19^ and by increasing the availability of contextual information, participants rely less on recently formed memory representations and more on a generalized scene grammar to guide search^20^. In Experiment 2 we investigated if inconsistent object arrangement can impede repeated search performance even if the inconsistencies were self-generated and how episodic memory usage is modulated by global and local objects. The experimental factors in Experiment 2 were Consistency (consistent vs. inconsistent), Object type (global vs. local objects), and Room type (other vs. own). The results of the individual analysis procedures are summarized in Table 3.

**Table 3:** Results of the simple linear mixed-model for reaction times including estimated regression coefficients together with the *t* statistic, as well as a Tukey corrected break down of significant interactions (left columns).

	RT LMM			RT LMM * Trial	
	Estimate	*t*		Estimate	*t*
(Intercept)	0.051	0.836		0.050	0.875
Condition (con vs. incon)	−0.136	−7.119		−0.138	−7.341
Object type (global vs. local)	−0.144	−5.634		−0.143	−7.638
Room type (own vs. other)	0.063	3.055		0.062	3.056
Trial (centered)				−0.031	−7.098
Condition × Object type	−0.043	−2.278		−0.040	−2.135
Condition × Room type	−0.014	−0.740		−0.014	−0.738
Object type × Room type	−0.049	−2.566		−0.050	−2.661
Condition × Trial				0.004	0.994
Object type × Trial				0.009	2.075
Room type × Trial				−0.008	−1.847
Condition × Object type × Room type	−0.025	−1.292		−0.024	−1.295
Condition × Object type × Trial				0.012	2.704
Condition × Room type × Trial				0.003	0.715
Object type × Room type × Trial				−0.001	−0.002
Condition × Object type × Room Type × Trial				−0.001	−0.112
	**Tukey contrasts of LMM interactions**				
	**Estimate**	***z***	***p***		
con (global) vs. incon (global)	−0.358	−5.793	0.001		
con (global) vs. con (local)	−0.376	−5.933	0.001		
con (global) vs. incon (local)	−0.560	−8.772	0.001		
incon (global) vs. con (local)	−0.018	−0.277	0.993		
incon (global) vs. incon (local)	−0.202	−3.140	0.009		
con (local) vs. incon (local)	−0.185	−4.143	0.001		
other (global) vs. own (global)	0.027	0.431	0.973		
other (global) vs. other (local)	−0.387	−6.035	0.001		
other (global) vs. own (local)	−0.164	−2.499	0.060		
own (global) vs. other (local)	−0.414	−6.306	0.001		
own (global) vs. own (local)	−0.191	−3.005	0.014		
other (local) vs. own (local)	0.223	4.731	0.001		

On the right, the results of the LMM including Trial as an interacting covariate are depicted.

#### The impact of consistency and object type on object interaction

As in Experiment 1, participants first completed the build phase. Replicating the findings of Experiment 1, global objects were not significantly affected by Consistency, whereas inconsistent local objects were grabbed longer compared to consistent ones type (Supplementary Figure 1 and Supplementary Table 1). Global objects were handled earlier during the trial than local ones. This constitutes a full replication of the experimental effects for grab duration in Experiment 1.

#### Search phase – assessing location memory and repeated search guidance

In the *search phase*, participants had to search for all objects within a room, before starting to search in the next one. Participants were not informed that they would search through rooms that they had built themselves (Room type = own) or search for objects in rooms built by participants from Experiment 1 (Room type = other).

**Figure 3:**
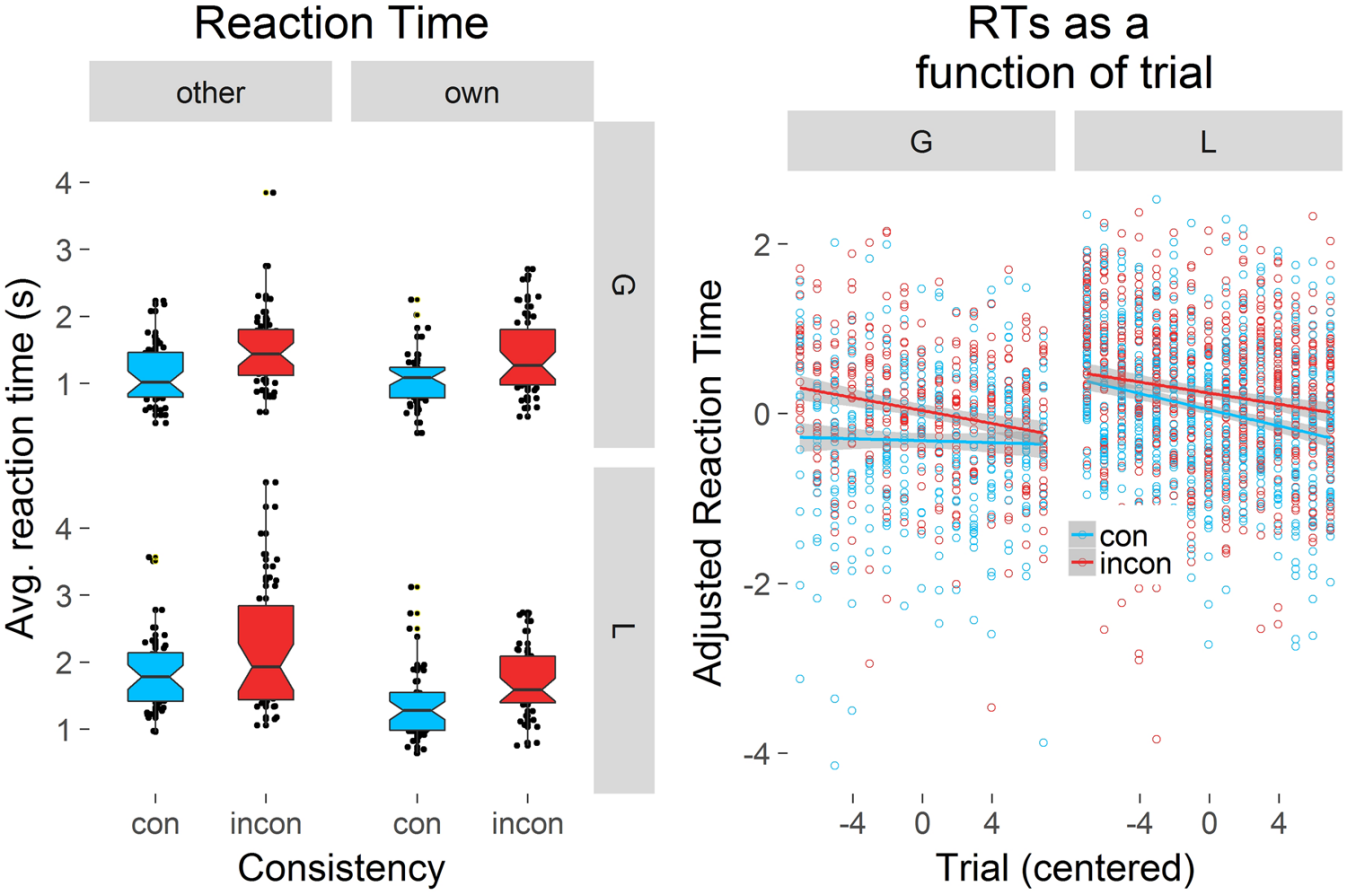
The effects of Consistency (consistent = con in blue vs. inconsistent = incon in red), Object type (global = G vs. local = L) and Room type (other vs. own) on RTs (left). Lower values indicate better location memory performance. The central mark is the median of each boxplot. The notches indicate 95% confidence intervals for the medians. The right graph displays partial effects (thus “adjusted”). RT on the y-axis against centered trial count as a function of Object type (global = G vs. local = L) and Consistency (consistent = con vs. inconsistent = incon). Shaded areas represent 95% confidence intervals.

Error trials were removed from the analysis, leading to the exclusion of 3.8% of the data. Reaction times (RTs) were faster for targets in the consistent, compared to inconsistent condition (Fig. 3, left). There also were significant main effects of Object type, with global objects being found faster than local, and Room type – RTs for own rooms were faster than for other rooms. There also was a significant interaction between Condition and Object type and a significant interaction between Room type and Object type, with an effect of Room type on local, but not on global objects. That is, local objects were found faster in own rooms than in other rooms, but this was not true for global objects.

To investigate if repeatedly searching through the same environment speeded search times, we included Trial (centered) as an interacting covariate (Fig. 3, right). The inclusion of the covariate did not alter the significance of the main effects from the previous model (Table 3). There was a significant main effect of Trial, as well as a significant interaction between Object type and Trial, and a significant three-way interaction between Condition, Object type, and Trial on search times. As can be seen in Fig. 3 (right), RTs for local objects improved to a greater degree than for global objects (steeper slopes) with every consecutive search within the same environment. RTs for inconsistent global objects became faster in comparison to consistent ones.

### The grammar of scenes

As a final step‚ we addressed the generative process of spatial arrangements, comparing that to the generation of words. Uncovering similarities between scene and word generation could provide first hints towards possible commonalities between scenes and language. The difference in the location memory baseline estimate between consistent and inconsistent global objects from Experiment 1 confirms common intuition that the predictions and knowledge of global scene structure are more similar when building a predictable rather than an unpredictable environment (see also Supplementary Figure 3).

**Figure 4:**
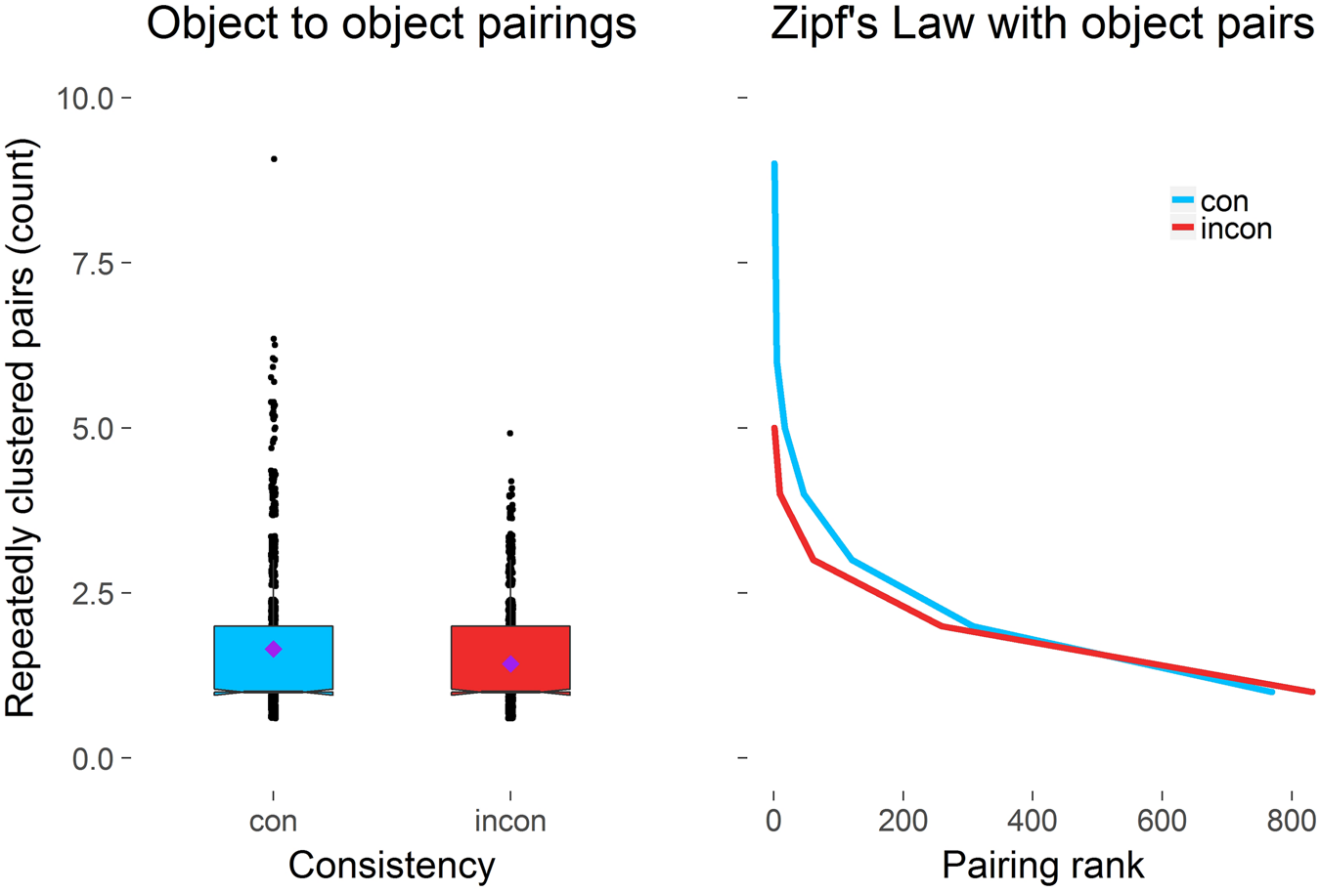
The effects of Consistency (consistent = con vs. inconsistent = incon) on the count of repeatedly clustered object pairs across rooms and participants (left). The central mark is the median of each boxplot. The notches indicate 95% confidence intervals for the medians. The purple diamonds mark the mean count per condition. The right graph displays the count of repeatedly clustered object pairs on the y-axis against the frequency-rank of each pair on the x-axis as a function of Consistency (consistent = con vs. inconsistent = incon). Shaded areas represent 95% confidence intervals.

In order to further investigate common predictions of scene structure and object-to-object relationships, we combined the data from the build phases of both experiments and calculated the closest local neighbor to each object for all objects in each room, by using the three-dimensional coordinates of the objects center (Fig. 4, left). We then counted all repeating object-to-object pairings across participants within rooms. For instance, both participant 1 and 6 had placed the toothbrush in close proximity to the sink; this would therefore be counted as a repeated pairing. A generalized linear mixed-model with a Poisson error distribution was used to model the count of repeatedly clustered object pairs as a function of Consistency. Consistency significantly predicted the amount of repeated pairs, *ß* = 0.073, SE = 0.167, *z* = 4.39, *p* < 0.001. Across rooms and participants, the same objects were clustered together more frequently in the consistent (e.g., shampoo as the closest neighbor to bathtub) compared to the inconsistent condition (e.g., handbag being the closest to pillow). This holds true across participants, indicating these pairings may be a part of some broader scene knowledge – or scene grammar – which we adhere to when constructing meaningful scene contexts.

The left graph of Fig. 4 not only demonstrates a higher count of frequently repeated object clusters, it also hints towards a difference in the distribution of object pairings. The spread in the consistent condition is much larger, with very *few* pairs being clustered together extremely frequently – e.g. oven and pot was paired together nine of possible ten times. To evaluate how generic this self-generated behavior is, we fitted the consistent and inconsistent pair count distributions to “near-Zipfian” distributions^37,38^ known from linguistics. These distributions are the modern incarnations of Zipf’s law^35,36^, a mathematically simple power law postulating that very *few* high frequency words (e.g., “a”, “the”, etc.) and *many* low frequency words (e.g. “badger”, “snout”, “quinoa”, etc.) will make up any given text. The right graph of Fig. 4 depicts the frequency of repeatedly clustered object pairs against the frequency-rank of each pair as a function of Consistency. Both conditions seem to follow a similar frequency/frequency rank relationship, yet with few, but highly-frequent object pairings the decline in the consistent condition was much more drastic. “Near-Zipfian” versions of the Zipf-Mandelbrot distribution^38^ fit both conditions best, but the distribution of the consistent object pairs, χ^2^ = 1.73 (5), *p* = 0.89 was better described than the distribution of inconsistent pairs, χ^2^ = 2.01 (3), *p* = 0.57.

## General Discussion

Knowing the rules and regularities underlying the arrangement of elements – here a *scene grammar* – has proven to be an essential feature in various tasks^2,4,20,39,40^. It has been proposed that these learned rules facilitate perceptual processes by generating predictions which can later be exploited^5,6^. Thus far, the vast majority of the literature has concentrated on how violations of scene grammar interrupt a given task. However, the process of generating rule-governed arrangements in scenes has largely been ignored, despite the fact that ongoing interactions with the external world are known to be an intricate part of human behavior^26,41–43^. In two experiments, we set out to describe how scene grammar within these self-generated scenes modulates the way in which we interact with objects and what, in turn, we remember about a scene, the essential building blocks of scene grammar as they develop during scene construction, and finally discover if there are systematic commonalities between occurrences of words in language and the spatial arrangement of scenes.

Violations of scene grammar can lead to slower and less accurate identification of objects^2,5,7^. Increasing the availability of contextual information, makes participants rely less on recently formed memory representations and more on a generalized scene grammar to guide search^20^. In our study, we asked participants to build scenes in a virtual reality environment in a way which either adhered or violated scene grammar. Subsequently, observers’ memory was probed through explicit recall (Experiment 1) or through a repeated search paradigm (Experiment 2). Our results show that contextual violations, even when self-inflicted, lead to a decrease in explicit memory performance and prolonged search times. This difference was beyond mere guessing performance, as participants performed significantly better than the between-subject baseline.

Further, with every subsequent search within the same environment reaction times decreased, showing that participants profited from repeatedly searching the same environment during this active task^44,45^, compared to searches in 2D^46–48^. However, the influence of scene grammar was mediated by the different building blocks of a scene: small/movable (local) and large/generally stationary (global) objects, which we have come to call “anchors”. While local objects benefited from memory in that they were found faster if the participant had arranged the room themselves, search for global objects was not speeded for rooms arranged by the participants themselves compared to rooms arranged by others. Moreover, global objects within consistent scenes did not benefit greatly from repeated search. Only within inconsistent scenes did search times for global objects improve with increasing familiarity of the environment. Moreover, constructing environments against one’s scene grammar led to longer processing time as measured by mean object grab time. This was true for local, but not for global objects. In addition, global objects were placed earlier in the trial.

Taken together, these results suggest that our scene knowledge applies differentially to the different building blocks of a scene: The global objects are used to establish the overall structure of a scene, as they are of primary importance for defining the shape of the space. They are placed early on during the construction and, importantly, may be used as “anchors” for spatial predictions regarding local objects (e.g. “I’ll place the book on the nightstand next to the bed”), because local objects tend to need global objects in order to have a well-defined place in the scene. The local objects, however, may be used to determine a more detailed scene grammar of the environment. During inconsistent trials, participants are specifically instructed to arrange scenes which do not follow their expectations, and it therefore takes them longer to decide where to put these local objects. In other words, there are limited spots to place a toothbrush within a consistent scene – close to the sink – however‚ there are countless locations one must decide between in an inconsistent scene. Our results demonstrate for the first time clear differences in goal directed behavior between global “anchors” and local objects.

Previous research has shown that participants provide strikingly similar objects when asked to name objects which are never found in a scene^49^. In our study‚ we calculated a between-participant baseline estimate for memory performance by comparing the location of each object placed in a room by one participant with the one placed by another. This provided us with first insights to commonalities between participants (see Supplementary Figure 3 for additional analysis). The significant interaction between object Consistency and Object type revealed that knowledge of *global* scene structure is more similar between participants when building a predictable rather than an unpredictable environment. This, however, is not the case for local objects, suggesting that while it takes more time to decide on the location of local objects in inconsistent rooms, there is still a strong consistency amongst different people regarding the locations which are finally chosen. In order to test the degree to which people implicitly rely on rule-governed representations of scenes we calculated the closest neighboring local object for each object in a room and subsequently checked the frequency with which this pairing repeated among all other subjects. For example, if participant 1 had placed the mouse close to the PC, how many other subjects did the same? Overall, objects were paired more frequently among the participants in the consistent condition compared to the inconsistent condition, suggesting once again that scenes are constructed according to a set of implicit universal rules shared across people. Moreover, we found that “near-Zipfian” distributions^37,38^ – which are usually found for the frequency of word occurrences in natural languages – described the frequency distributions of object pairing better in the consistent compared to the inconsistent condition. In the current study, there was one object pair – oven and pot – which was placed in close proximity by nearly all of the subjects (90%), a few pairs which were placed next to each other slightly less frequently, and finally the majority of the pairings were only placed together by one subject each. In other words, a few rules – or in this case object pairings – seem to be widely accepted, while the great majority of objects are arranged in less systematic ways. Such power laws have been found in a variety of scientific fields^50^, yet explaining them is not trivial. A recent proposal states that human memory might be responsible for a power law for word frequencies^37^, as memory *decay* ^51,52^ and *search* through memory for a particular item^53,54^ can be described by power laws. In light of this view, it could be argued that object pairings in the consistent condition follow “near-Zipfian” distributions more closely, as they are pooled from a life-time of learning. This contrasts with the inconsistent case, as violating scene grammar goes directly against learned spatial regularities. Further investigations of possible commonalities between language and vision might be able to elucidate the nature of those cognitive processes that make us humans as efficient as we are.

In sum, we provide evidence that scene grammar not only influences how we interact with objects and what we remember about a scene, but we also for the first time reveal some of the important building blocks of scene grammar – i.e. global “anchors” and local objects - and the differential roles they take on during the construction of a natural environment. Finally, our results suggest that cognitive processes underlying the construction of language by the use of word on the one hand and the construction of scenes by use of objects on the other seem to produce similar distributions, which could be an important step towards a better understanding of the commonalities between the use of both language and naturalistic environments.

## General Methods

### Participants

Ten participants per experiment (Exp1: mean age = 19.7, range = 19–21, 9 female, 1 left-handed; Exp2: mean age = 23, range = 19–34, 7 female, all right-handed) were recruited at the Goethe University Frankfurt. All participants had normal or corrected-to-normal vision, were volunteers receiving course credit, and gave informed consent. All methods were carried out in accordance with the guidelines and regulations of the granting body. The experimental protocol was approved by the Ethics Commission of the Department of Psychology.

### Apparatus

The participants wore a HTC Vive head mounted display (HMD). The HMD has two 1080 × 1200 pixel resolution screens, with a refresh rate of 90 Hz and approx. 100° × 110° field of view. Motion tracking with a sub-millimeter precision is achieved via two base stations emitting structured infrared light. The HMD includes 37 photosensors and the wireless motion tracked controller which participants used to interact with objects in the virtual environment includes 24 photosensors. Participants’ eye movements were recorded using the SMI eye tracking integration for the HTC Vive with an accuracy of approx. 0.2° at the refresh rate of the HMD - 90 Hz. The virtual environment was presented and rendered with Vizard 5 by WorldViz on a high-performance laptop running Windows 10.

### Environment

Sixteen virtual rooms with identical floor plans (each approx. 3 × 3 m) of four different categories (living room, kitchen, bedroom, & bathroom) were used in both experiments. One additional room (gym) was used for practice trials. Each room had one blue wall onto which the instructions were presented and which functioned as a reference point in the virtual environment. All objects were carefully chosen to represent the semantic category of the room they were found in. Each room contained an a priori defined set of 10 *local* and 5 *global* objects (overall 240 objects). Global objects were defined as objects which are usually static in a room (rarely move), provide spatial layout, and are typically large (e.g., shower, bed, fridge, etc.). All other objects in a room were considered to be local objects – objects we usually act upon.

### Procedure

Upon arrival, participants were familiarized with the HMD, the wireless controller, and the lab space. They were informed that a virtual light grid would appear whenever they were in proximity of physical obstructions, such as walls or tables. Additionally, they completed a short practice session during which they were accustomed to both the HMD and the testing procedure.

**Figure 5:**
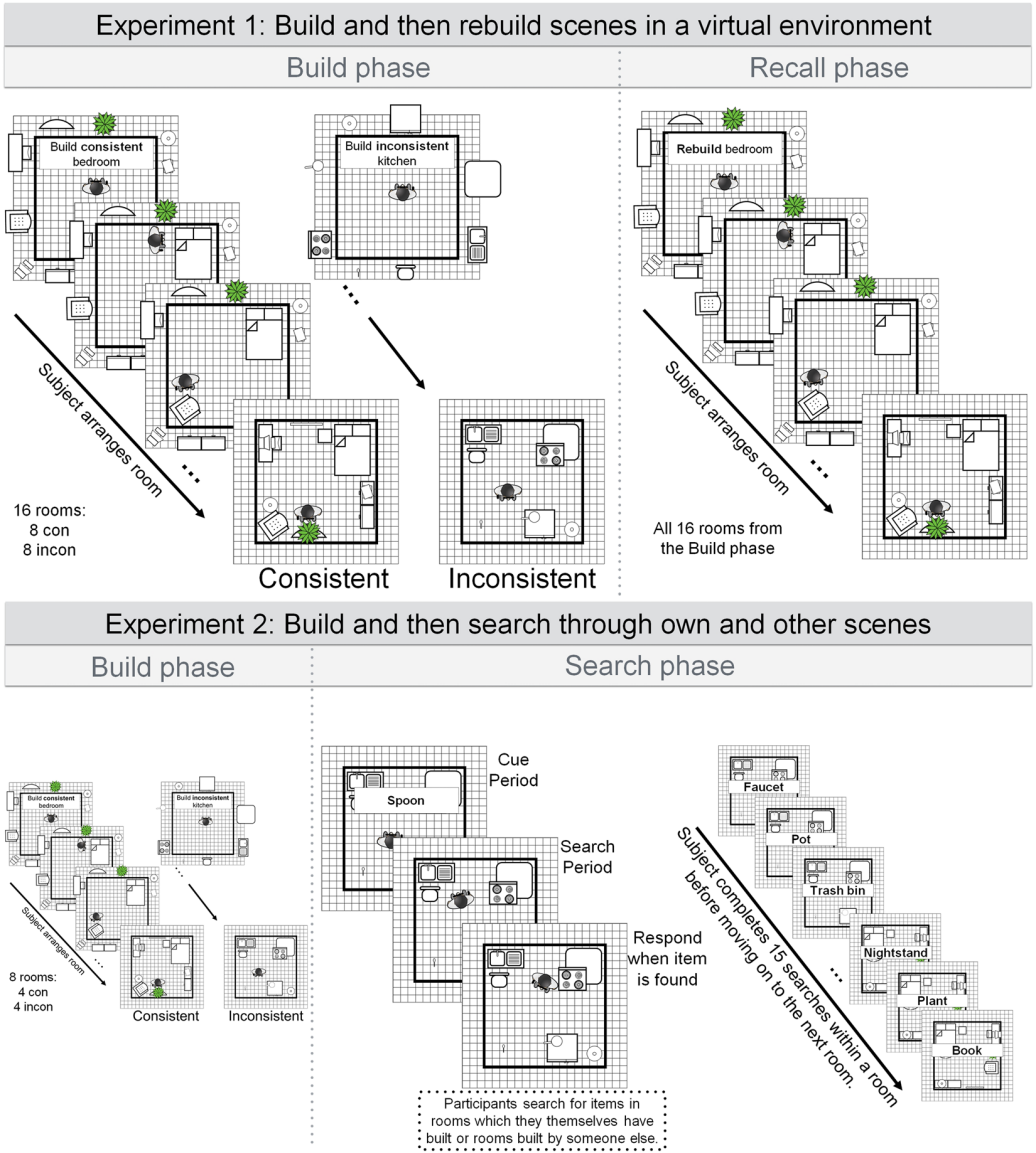
The experimental procedure of Experiment 1 (top) and Experiment 2 (bottom). The procedure of the build phase was identical for both experiments – participants were instructed to either arrange objects in a consistent or inconsistent fashion. In Experiment 1, a recall phase followed in which participants were instructed to rebuild the environments from the build phase. In Experiment 2, participants had to sequentially search for all objects in the rooms they had built, as well as rooms built by participants in Experiment 1.

In both experiments, the procedure consisted of two phases – the build phase and a test phase (recall in Experiment 1 and search in Experiment 2). During the *build phase* participants were instructed to arrange objects in each room either according (consistent condition) or against (inconsistent condition) their expectations regarding object locations (Fig. 5). Room assignment to the conditions was counterbalanced across participants. Each trial began with participants standing in the middle of an empty room facing the blue wall onto which the instructions were presented. Before the trial started the calibration procedure commenced and participants were informed whether the next room would need to be arranged in a consistent or inconsistent fashion. Subsequently 15 objects appeared, hovering one meter above the ground on a circular array with a radius of 1.5 m – the center being the center of the room where the participants stood. The assignment of the objects to the 15 possible locations was random. Participants could *grab* these objects by intersecting their controller with the objects’ surface and pressing the trigger button of their controller. This allowed participants to drag and drop objects throughout the virtual space. Subjects had become familiar with this procedure during practice. In both conditions, participants were instructed to avoid violating physics (e.g., not allowing objects to float in midair), make sure objects remain visible and reachable (e.g., not putting the bathtub on top of the comb), and place objects within the bounds of the room. Participants were instructed to place objects quickly and intuitively, without overthinking either the consistent or the inconsistent placement. Apart from these limitations, participants were free to arrange the rooms however they desired. Once the participants completed a room, they pressed the menu button on their controller to initiate the next trial. The procedure of the build phase was the same for both experiments, with the only difference being that participants had to arrange twice as many rooms in Experiment 1 (16 rooms) than in Experiment 2 (8 rooms). Video material of example trial procedures is available on (https://youtu.be/_VzaVPrnHOI; https://youtu.be/vHN75xIFdW4).

The test phase differed between experiments. In Experiment 1, after completing the arrangement of the 16 rooms, participants were informed of the surprise recall procedure that followed. In the *recall phase*, participants had to rebuild all the rooms in the same way as they had done during the build phase - they were instructed to place objects in the exact locations they had placed them before. Participants completed the rooms in the same order as they had during the first phase.

In Experiment 2, after the build phase, participants had to search for all objects within each room (*search phase*). Each trial began with the target cue being presented for 750ms in the center of the visual field. Subsequently, the participant had to find the target as fast and as accurately as possible. Participants were free to move and explore the environment in order to complete their task efficiently. Upon finding the target, the participants were instructed to fixate the target and press the touch pad button of the controller. Participants were not required to move towards or touch the target. Following the button press, the new target cue appeared after 500ms. Participants searched for all 15 objects within a room, before starting to search in the next one. Participants were not informed that they would search through rooms that they had built (Room type = own) or search for objects in rooms built by participants in Experiment 1 (Room type = other). Each participant in Experiment 2 searched through the rooms of the participant with the matching subject number from Experiment 1 (i.e., Participant Nr.4 – Exp2 matched with Participant Nr.4 – Exp1). The assignment of rooms to the own and other conditions, as well as to consistent/inconsistent conditions was counterbalanced across subjects. The order of object searches was randomized.

### Data Analysis

To analyze object interaction behavior, we recorded grab duration and order for each time an object was grabbed and released. This allowed us to calculate the mean grab duration per condition for each participant, as well as the summed grab duration for each object. Raw eye movement data was sampled at 90 Hz and written to file whenever an object was viewed. All samples on each object were summed in order to calculate summed gaze durations for each object as a function of the experimental conditions and participants.

To assess location memory performance in the recall task of Experiment 1, we calculated the distance in three-dimensional space between the original location of each object’s center and its location at recall. Reaction times in Experiment 2 were calculated as the time from the offset of the target cue until the participants pressed the response button.

For analyzing the effects in our data, linear mixed-effects models (LMMs) were run using the *lme4* package^55^ in the R statistical programming environment^56^. We chose the LMM approach as it allows between-subject and between-item variance to be estimated simultaneously and thus yields advantages over traditional F1\ F2 analysis of variance^57,58^. Repeated measures Analysis of Variances (ANOVAs) were conducted using the *ez* package^59^ when it was necessary to aggregate data.

All LMMs were fitted with the maximum likelihood criterion. All experimental factors consisted of two levels and sum contrasts were defined to analyze the critical comparisons. Participants and rooms were included in the models as random factors. In practice, models with random intercepts and slopes for all fixed effects often fail to converge or lead to overparameterization^60^. In order to produce models that converge on a stable solution and are properly supported by the data, we used a Principal Components Analysis (PCA) of the random-effects variance-covariance estimates for each fitted mixed-effects model to identify overparameterization^60^. Random slopes not supported by the PCA and not contributing significantly to the goodness of fit (likelihood ratio tests), were removed from the model. Details about the retained variance components of each model can be found in the final analysis scripts.

Following inspection of the distribution/residuals and the power coefficient output of the boxcox procedure^61,62^, all dependent variables were log-transformed in order to more closely approximate a normal distribution and meet LMM assumptions. We report regression coefficients with the *t* statistic and apply the two-tailed criterion (|*t*| ≥ 1.96), corresponding to a 5% error criterion for significance. To break down significant interactions, the *lsmeans* package^63^ was used to obtain least-squares means and perform Tukey adjusted comparisons of factor levels.

Figures depicting the influence of covariates in this paper are based on partial effects created with the *remef* package^64,65^ and were programmed with *ggplot2* ^66^. Figures with partial effects computed from model parameters reproduce the estimated statistical effects and allow for a straightforward interpretation of the results.

### Data availability

Raw data, preprocessing scripts, and preprocessed data as well the final analysis scripts can be found under: https://www.dropbox.com/sh/4yrtvdls65vbybi/AABzMzSn-hdT9wkpVyQluXS5a?dl=0 (upon acceptance an upload to https://osf.io/p7346/ will follow).

## Electronic supplementary material

Supplementary Materials
