# Supplementary material for: Scene grammar shapes the way we interact with objects, strengthens memories, and speeds search
Source: Sci Rep. 2017 Nov 28;7:16471. doi: 10.1038/s41598-017-16739-x (PMC5705766; doi:10.1038/s41598-017-16739-x)
Supplement: Supplementary file 1 — Supplementary Materials [file 41598_2017_16739_MOESM1_ESM.pdf]

Scene grammar shapes the way we interact with objects, strengthens memories, and speeds search.

Dejan Draschkow & Melissa L.-H. Võ

Scene Grammar Lab, Johann Wolfgang Goethe-Universität, Frankfurt, Germany

Corresponding author contact information:

Dejan Draschkow

Scene Grammar Lab
Department of Cognitive Psychology
Goethe University Frankfurt
PEG, Room 5.G105
Theodor-W.-Adorno-Platz 6
60323 Frankfurt am Main, Germany

Phone: +49 (0)69 798 35310
Mail: draschkow@psych.uni-frankfurt.de
Web: www.draschkow.com; www.SceneGrammarLab.com

Keywords: *Virtual reality; Scene grammar; Episodic memory; Semantic memory; Object interaction; Object handling; Visual search; Eye movements*

Supplementary Materials

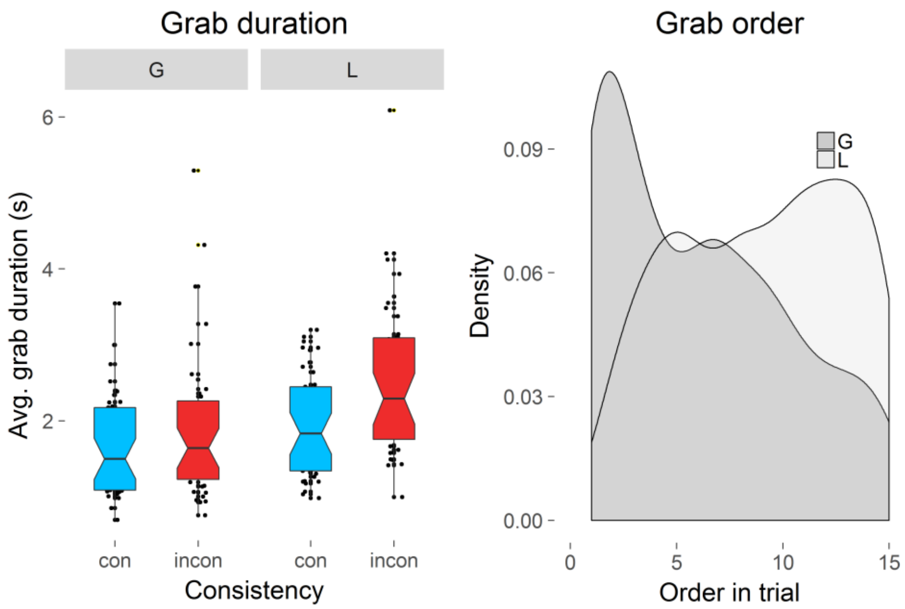

Supplementary Figure 1: The effects in Experiment 2 of Consistency (consistent = con vs. inconsistent = incon), Object type (global =G vs. local = L) and experimental Phase (build vs. recall) on the average grab duration (left). The central mark is the median of each boxplot. The notches indicate 95% confidence intervals for the medians. The right graph depicts computed density estimates (Gaussian smoothing kernel) (y-axis) for first object grabs during a trial (x-axis) as a function of Object type (global =G vs. local = L).

Supplementary Table 1: Results of the Experiment 2 LMM for grab duration including estimated regression coefficients together with the *t* statistic, as well as a Tukey corrected break down of significant interactions (left columns). On the right, the statistics of the ANOVA for mean grab order are listed.

	**Grab duration LMM**			**Grab order ANOVA**		
	Estimate	*t*	df	*F*	*p*	η^2_G
**(Intercept)**	0.120	1.280				
**Condition (con vs. incon)**	-0.080	-6.583	1,9	0.916	0.363	0.003
**Object type (global vs. local)**	-0.095	-4.036	1,9	29.000	0.001	0.684
**Condition × Object type**	0.054	4.506	1,9	0.793	0.396	0.018
	**Tukey contrasts of LMM interaction**					
	Estimate	*z*	*p*			
**con (global) vs. incon (global)**	-0.052	-1.543	0.412			
**con (global) vs. con (local)**	-0.083	-1.628	0.363			
**con (global) vs. incon (local)**	-0.350	-6.578	0.001			
**incon (global) vs. con (local)**	-0.031	-0.586	0.936			
**incon (global) vs. incon (local)**	-0.298	-5.444	0.001			
**con (local) vs. incon (local)**	-0.267	-7.778	0.001			

The effect of grab duration on reaction times

To investigate the predictive properties of grab and gaze duration, we included them as covariates in a LMM (Supplementary Figure 2). The LMM was performed on a subset of the data – only rooms which participants actually built were included. Visual exposure duration was not a significant predictor of search times. Even though object interaction time did not predict search times either, longer interaction time with the objects neutralized the reaction time benefit of consistent compared to inconsistent environments.

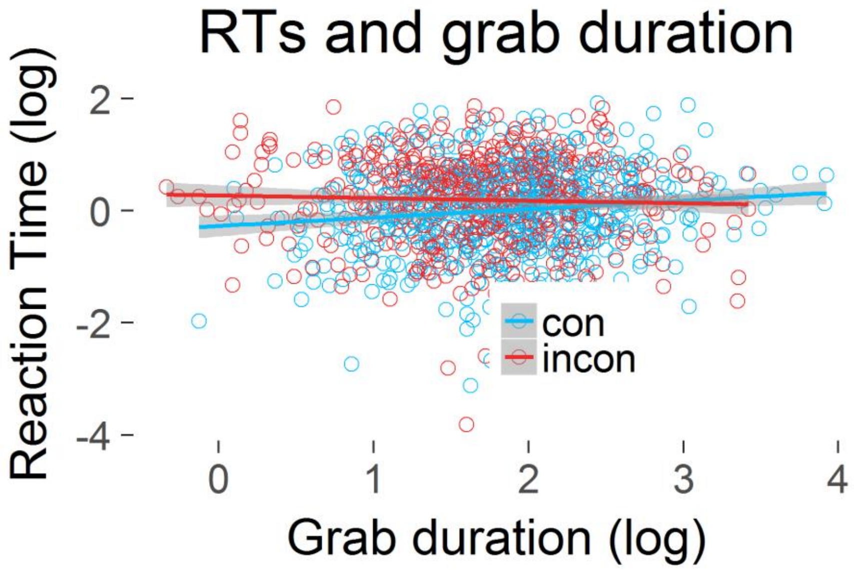

Supplementary Figure 2: The graph displays partial effects. Reaction times on the y-axis against log transformed grab duration per object on the x-axis as a function of Consistency (consistent = con vs. inconsistent = incon). Shaded areas represent 95 % confidence intervals.

Supplementary Table 2: Results of the Experiment 2 LMM with covariates for search times including estimated regression coefficients together with the *t* statistic.

	**RT LMM * covariates**	
	Estimate	*t*
**(Intercept)**	-0.029	-0.259
**Condition (con vs. incon)**	-0.270	-3.084
**Object type (global vs. local)**	0.068	0.766
**Grab duration (log)**	0.052	0.989
**Gaze duration (log)**	-0.063	-1.589
**Condition × Object type**	0.051	0.587
**Condition × Grab duration (log)**	0.098	2.286
**Object type × Grab duration (log)**	-0.066	-1.550
**Condition × Object type × Grab duration (log)**	-0.044	-1.043

Inter-subject similarity in object placement

To investigate the generative contextual consistency of object placement within virtual environments we calculated the similarity of spatial arrangements between participants in both experiments (Supplementary Figure 3). We computed the per participant distance between each object to all other objects in a room and the Spearmen rank order correlation between those distances. The resulting correlation matrix was averaged yielding a single correlation value for each room in each condition. Room arrangements were more similar to each other across participants in the consistent compared to the inconsistent condition, $F(1, 15) = 41.7$, $p < 0.001$, $\eta^2_G = 0.53$.

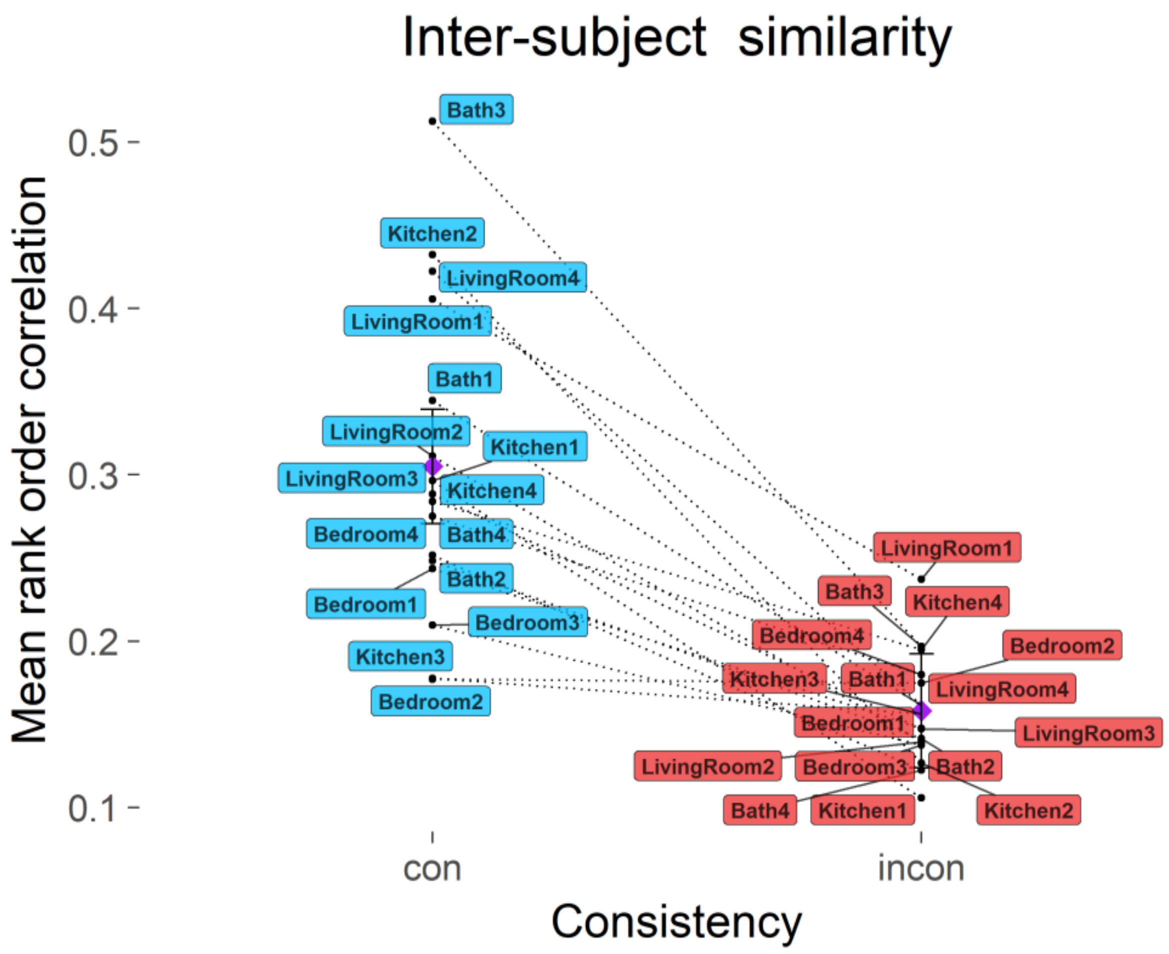

Supplementary Figure 3: The effect of Consistency (consistent = con vs. inconsistent = incon) on the average rank order correlation (y-axis) for each room. The purple diamonds mark the mean values per condition and the error bars represent 95% confidence intervals. Rooms are connected with dotted lines to represent the directionality of the difference between the Consistency conditions.
